# Supplementary material for: Hsa_circ_0056686, derived from cancer-associated fibroblasts, promotes cell proliferation and suppresses apoptosis in uterine leiomyoma through inhibiting endoplasmic reticulum stress
Source: PLoS One. 2022 Apr 7;17(4):e0266374. doi: 10.1371/journal.pone.0266374 (PMC8989227; doi:10.1371/journal.pone.0266374)
Supplement: S1 Raw images — (PDF) [file pone.0266374.s001.pdf]

Supporting information

The full membrane images of all protein involved in the manuscript.

The protein bands were visualized by using the Enhanced chemiluminescence reagents (Millipore, MA, USA). The expression of relative protein was obtained by the gray value ratio of the target protein to the internal reference GAPDH and analyzed with ImageJ software (National Institutes of Health, Bethesda, MA, USA).

Figure 4A

GRP78

① ② ③ ④

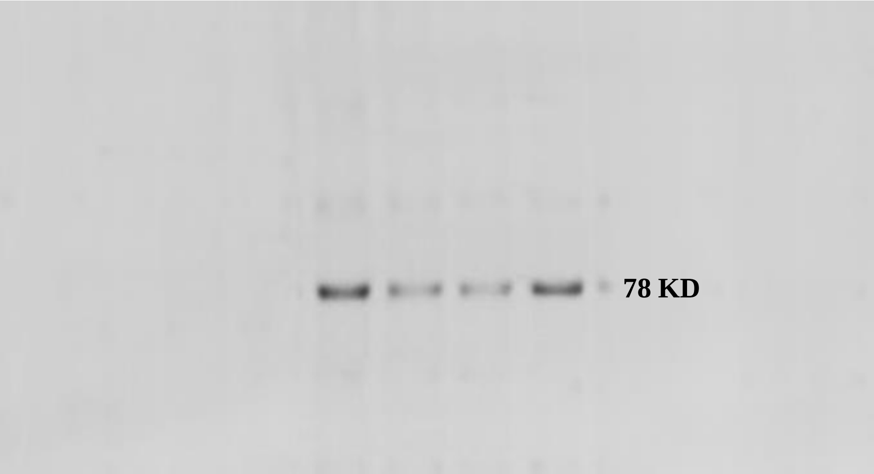

① **control** ② **CAFs-CM** ③ **CAFs$^{sh\text{-}NC}$-CM** ④ **CAFs$^{sh\text{-}circ_0056886}$-CM**

Figure 4A

CHOP

① ② ③ ④

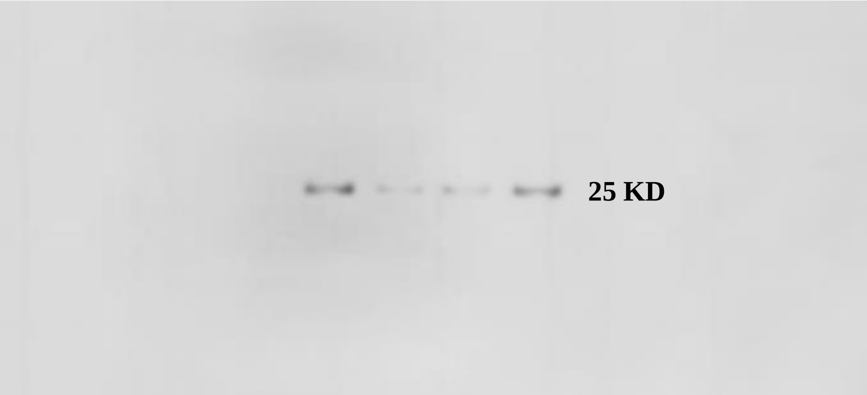

① **control** ② **CAFs-CM** ③ **CAFs$^{sh\text{-}NC}$-CM** ④ **CAFs$^{sh\text{-}circ_0056886}$-CM**

Figure 4A

ATF6

① ② ③ ④

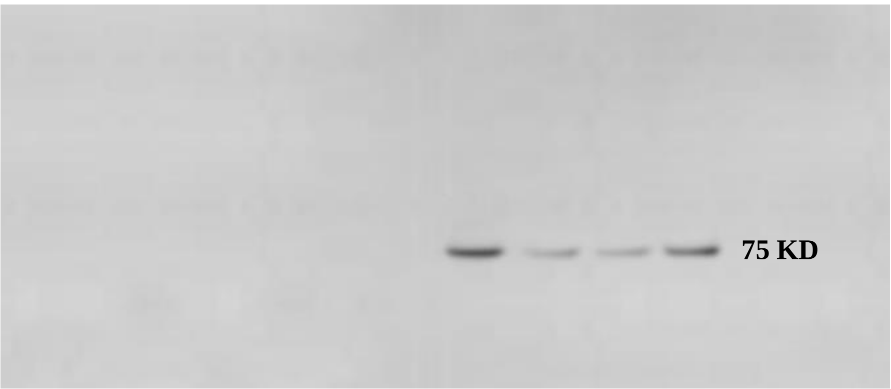

① **control** ② **CAFs-CM** ③ **CAFs$^{sh\text{-}NC}$-CM** ④ **CAFs$^{sh\text{-}circ_0056886}$-CM**

Figure 4A

GAPDH

① ② ③ ④

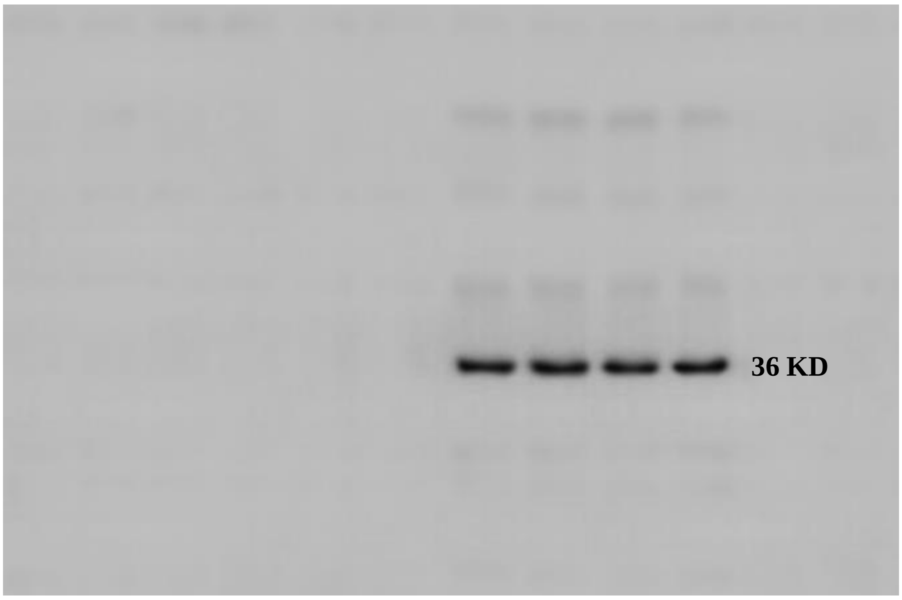

① **control** ② **CAFs-CM** ③ **CAFs$^{sh\text{-}NC}$-CM** ④ **CAFs$^{sh\text{-}circ_0056886}$-CM**

Figure 4B

Cleaved-caspase3

① ② ③ ④

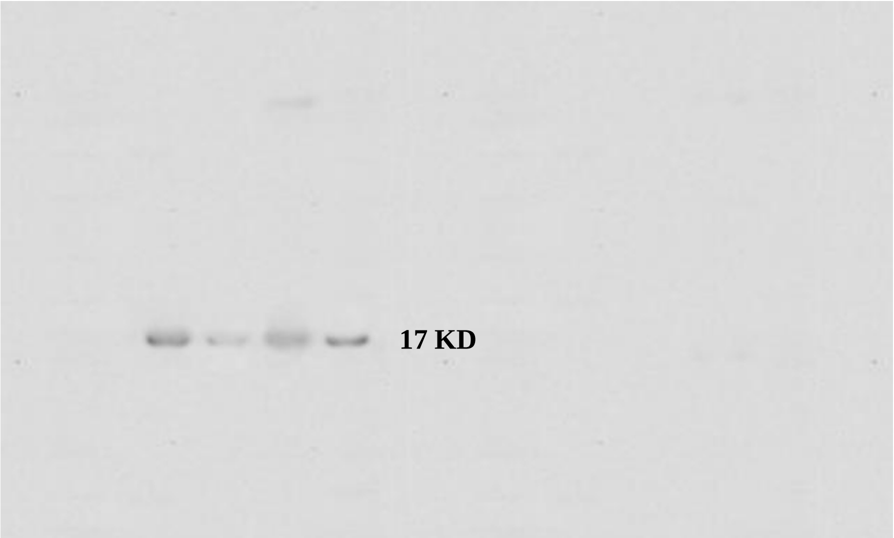

① **control** ② **CAFs-CM** ③ **CAFs$^{sh\text{-}NC}$-CM** ④ **CAFs$^{sh\text{-}circ_0056886}$-CM**

Figure 4B

BCL-2

① ② ③ ④

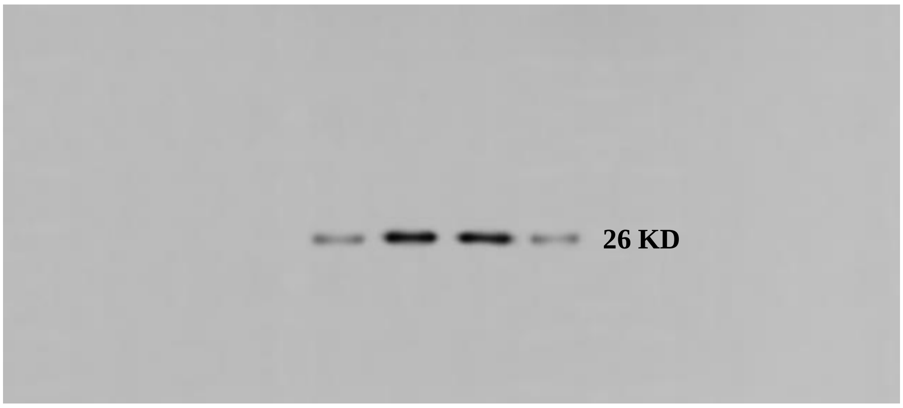

① **control** ② **CAFs-CM** ③ **CAFs$^{sh\text{-}NC}$-CM** ④ **CAFs$^{sh\text{-}circ_0056886}$-CM**

Figure 4B

GAPDH

① ② ③ ④

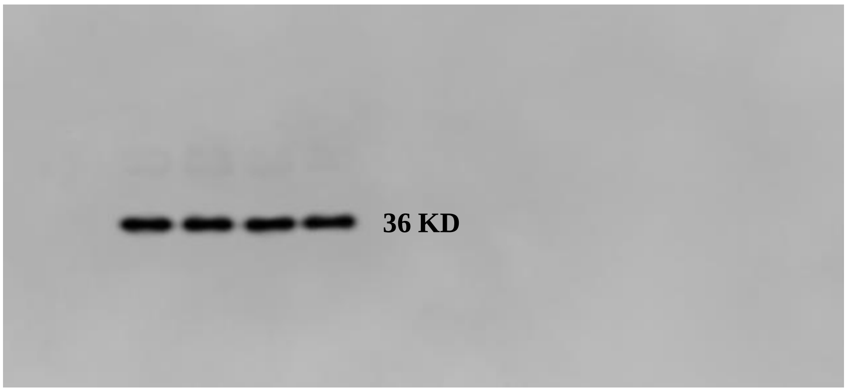

① **control** ② **CAFs-CM** ③ **CAFs$^{\text{sh-NC}}$-CM** ④ **CAFs$^{\text{sh-circ_0056886}}$-CM**

Figure 4C

COL1A1

① ② ③ ④

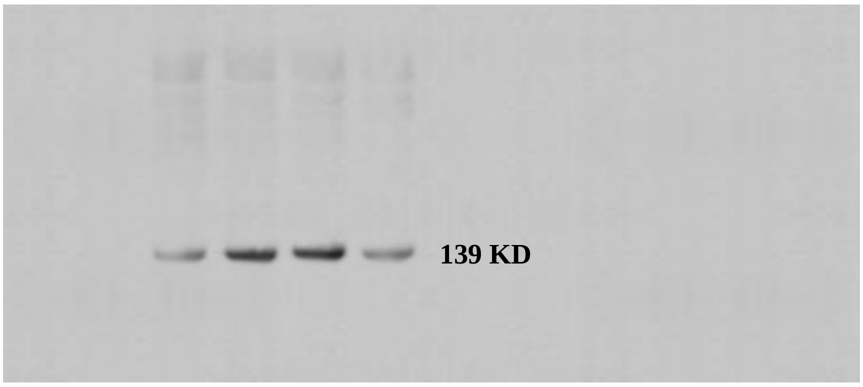

① **control** ② **CAFs-CM** ③ **CAFs$^{\text{sh-NC}}$-CM** ④ **CAFs$^{\text{sh-circ_0056886}}$-CM**

Figure 4C

COL1A2

① ② ③ ④

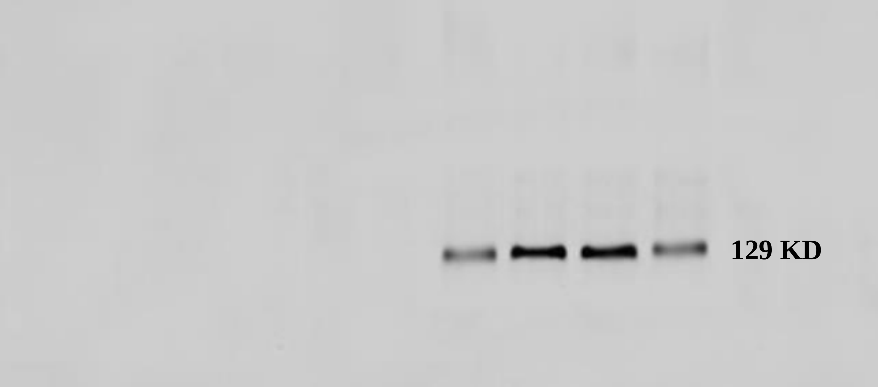

① **control** ② **CAFs-CM** ③ **CAFs$^{sh\text{-}NC}$-CM** ④ **CAFs$^{sh\text{-}circ_0056886}$-CM**

Figure 4C

COL3A1

① ② ③ ④

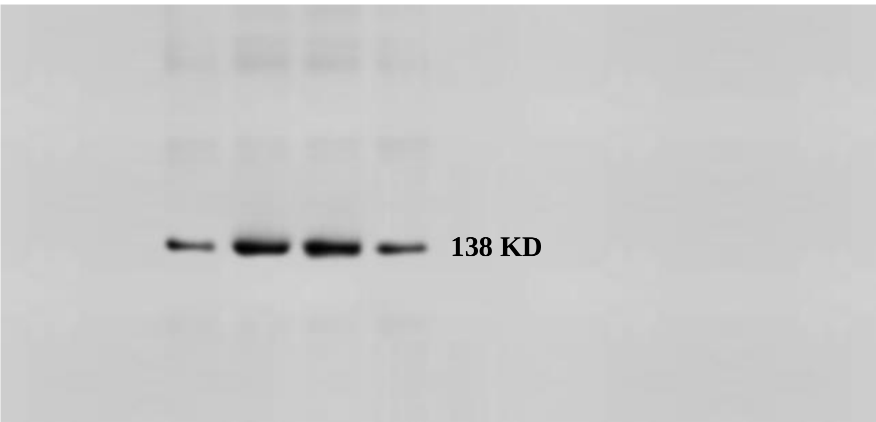

① **control** ② **CAFs-CM** ③ **CAFs$^{sh\text{-}NC}$-CM** ④ **CAFs$^{sh\text{-}circ_0056886}$-CM**

Figure 4C

GAPDH

① ② ③ ④

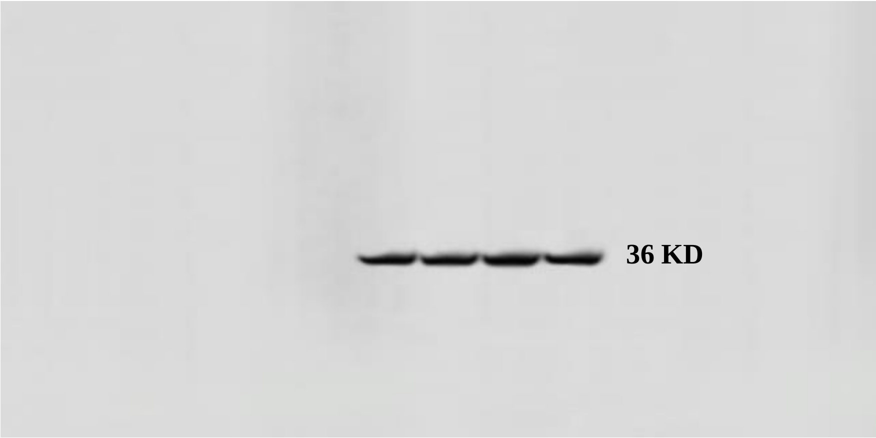

① **control** ② **CAFs-CM** ③ **CAFs$^{sh\text{-}NC}$-CM** ④ **CAFs$^{sh\text{-}circ_0056886}$-CM**

Figure 5B

GRP78

① ② ③ ④ ⑤

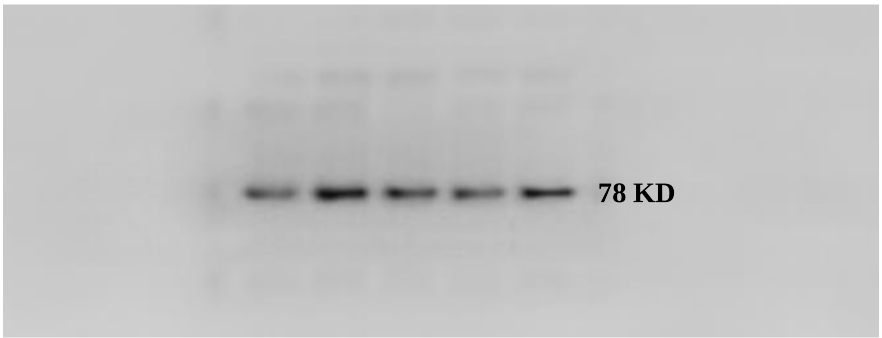

① **control** ② **Brefeldin A** ③ **Brefeldin A+CAFs-CM** ④ **Brefeldin A+CAFs$^{sh\text{-}NC}$-CM**

⑤ **Brefeldin A+CAFs$^{sh\text{-}circ_0056886}$-CM**

Figure 5B

CHOP

① ② ③ ④ ⑤

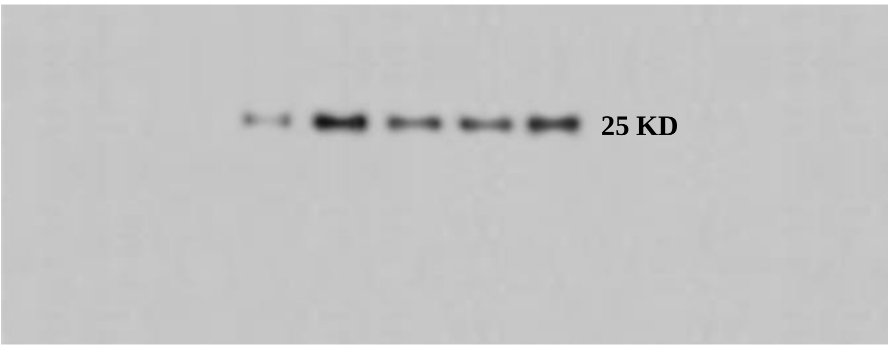

① control ② Brefeldin A ③ Brefeldin A+CAFs-CM ④ Brefeldin A+CAFs^{sh-NC}-CM

⑤ Brefeldin A+CAFs$^{sh-circ_0056886}$-CM

Figure 5B

ATF6

① ② ③ ④ ⑤

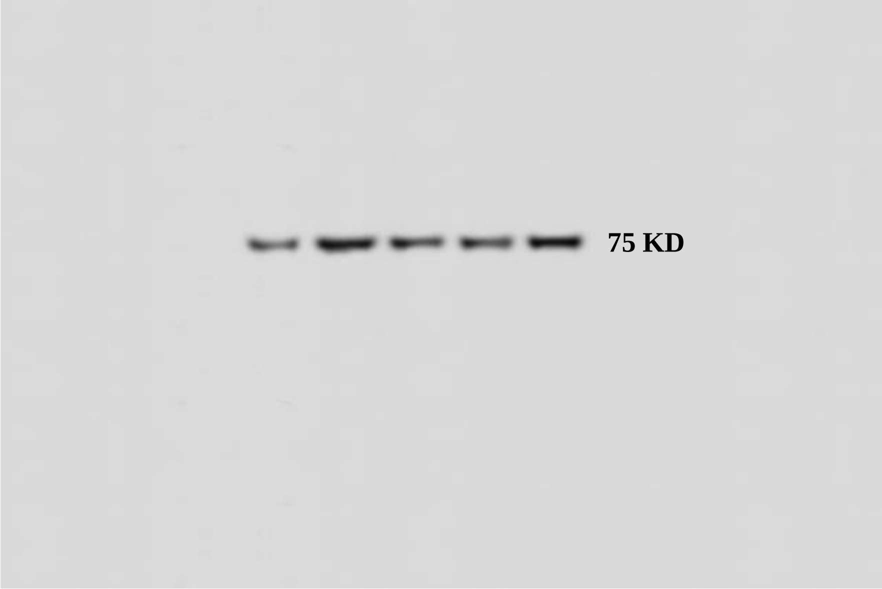

① control ② Brefeldin A ③ Brefeldin A+CAFs-CM ④ Brefeldin A+CAFs^{sh-NC}-CM

⑤ Brefeldin A+CAFs$^{sh-circ_0056886}$-CM

Figure 5B

GAPDH

① ② ③ ④ ⑤

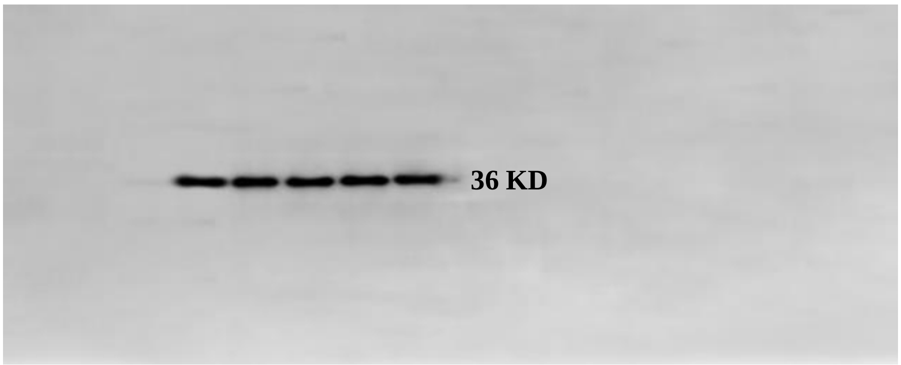

① control ② Brefeldin A ③ Brefeldin A+CAFs-CM ④ Brefeldin A+CAFs$^{sh\text{-}NC}$-CM ⑤ Brefeldin A+CAFs$^{sh\text{-}circ_0056886}$-CM

Figure 7C

Cleaved-caspase3

① ② ③ ④ ⑤ ⑥

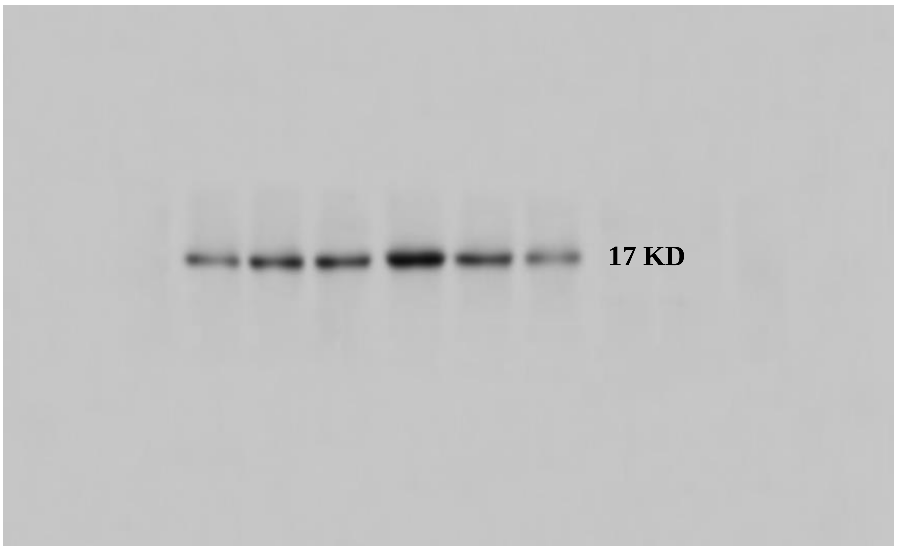

① CAFs$^{sh\text{-}NC}$-CM ② CAFs$^{sh\text{-}hsa_circ_0056686}$-CM ③ CAFs$^{sh\text{-}hsa_circ_0056686}$-CM+NC mimic ④ CAFs$^{sh\text{-}hsa_circ_0056686\text{-}CM}$+miR-515-5p mimic ⑤ CAFs$^{sh\text{-}hsa_circ_0056686}$-CM+NC inhibitor ⑥ CAFs$^{sh\text{-}hsa_circ_0056686\text{-}CM}$+miR-515-5p inhibitor

Figure 7C

BCL-2

① ② ③ ④ ⑤ ⑥

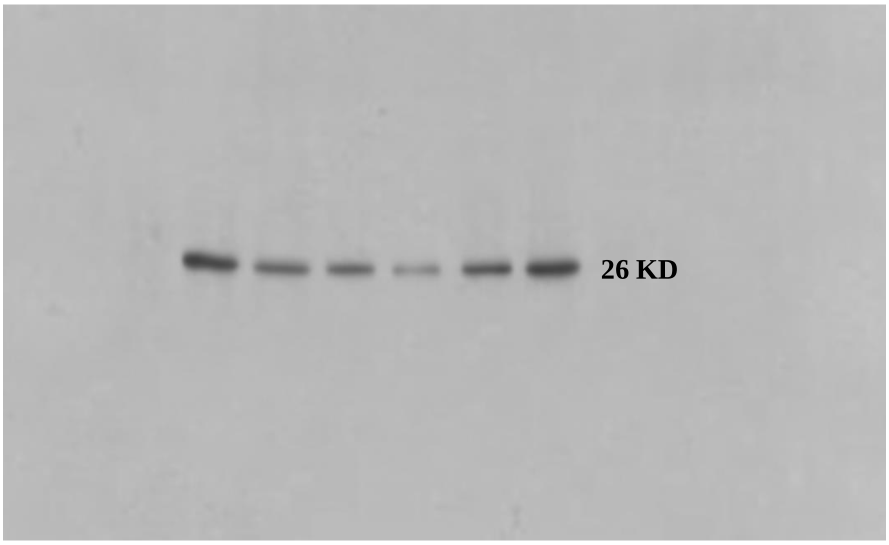

① CAFs$^{\text{sh-NC}}$-CM ② CAFs$^{\text{sh-hsa_circ_0056686}}$-CM ③ CAFs$^{\text{sh-hsa_circ_0056686}}$-CM+NC mimic ④ CAFs$^{\text{sh-hsa_circ_0056686-CM}}$+miR-515-5p mimic ⑤ CAFs$^{\text{sh-hsa_circ_0056686}}$-CM+NC inhibitor ⑥ CAFs$^{\text{sh-hsa_circ_0056686-CM}}$+miR-515-5p inhibitor

Figure 7C

GAPDH

① ② ③ ④ ⑤ ⑥

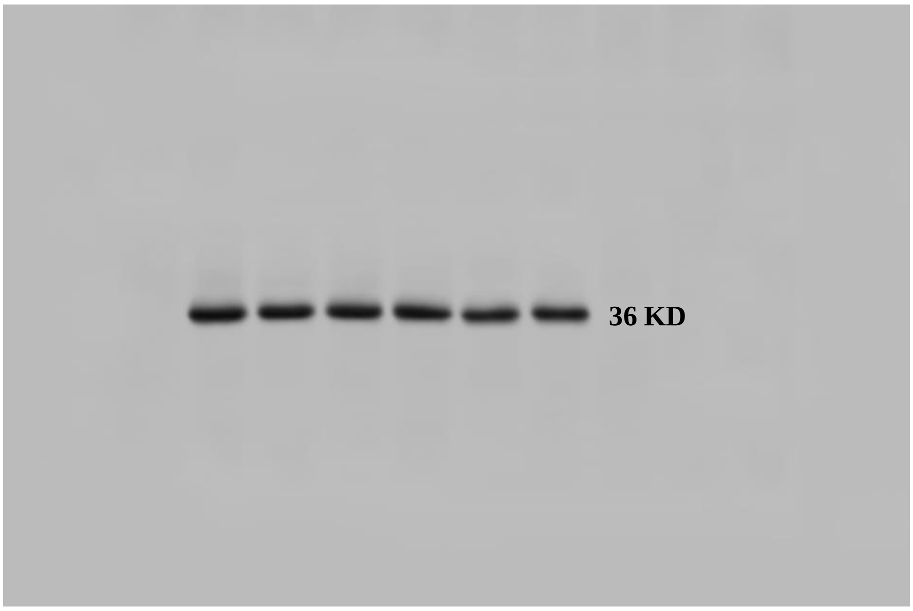

① CAFs$^{\text{sh-NC}}$-CM ② CAFs$^{\text{sh-hsa_circ_0056686}}$-CM ③ CAFs$^{\text{sh-hsa_circ_0056686}}$-CM+NC mimic

④ CAFs$^{\text{sh-hsa_circ_0056686-CM}}$+miR-515-5p mimic ⑤ CAFs$^{\text{sh-hsa_circ_0056686}}$-CM+NC inhibitor

⑥ CAFs$^{\text{sh-hsa_circ_0056686-CM}}$+miR-515-5p inhibitor

Figure 7D

GRP78

① ② ③ ④ ⑤ ⑥

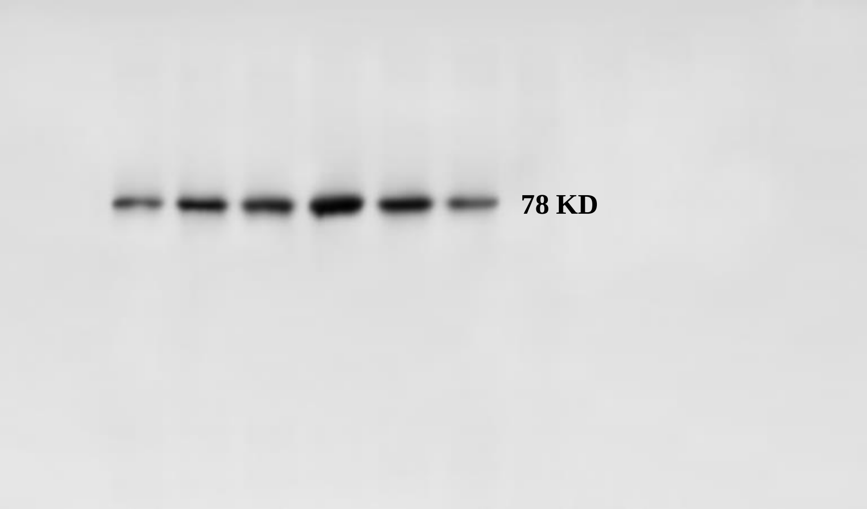

① CAFs$^{\text{sh-NC}}$-CM ② CAFs$^{\text{sh-hsa_circ_0056686}}$-CM ③ CAFs$^{\text{sh-hsa_circ_0056686}}$-CM+NC mimic

④ CAFs$^{\text{sh-hsa_circ_0056686-CM}}$+miR-515-5p mimic ⑤ CAFs$^{\text{sh-hsa_circ_0056686}}$-CM+NC inhibitor

⑥ CAFs$^{\text{sh-hsa_circ_0056686-CM}}$+miR-515-5p inhibitor

Figure 7D

CHOP

① ② ③ ④ ⑤ ⑥

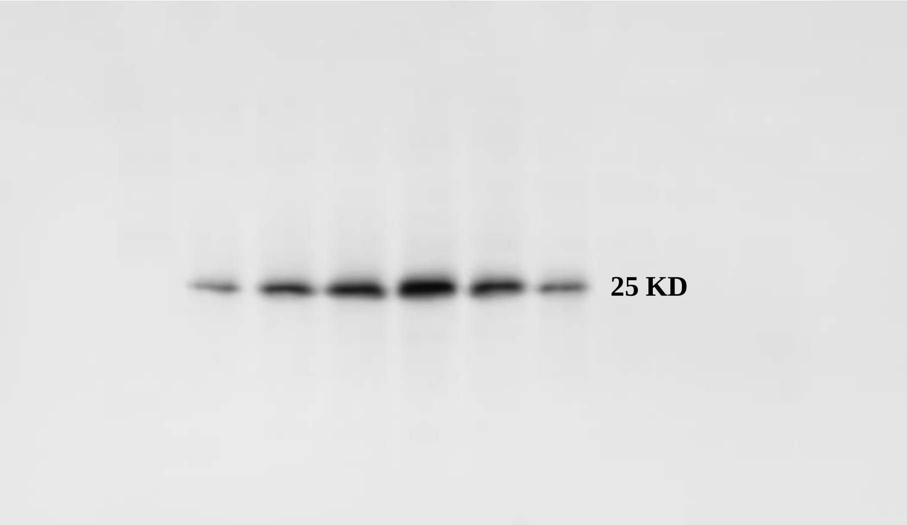

① $\text{CAFs}^{\text{sh-NC}}$-CM ② $\text{CAFs}^{\text{sh-hsa\_circ\_0056686}}$-CM ③ $\text{CAFs}^{\text{sh-hsa\_circ\_0056686}}$-CM+NC mimic

④ $\text{CAFs}^{\text{sh-hsa\_circ\_0056686-CM}}$+miR-515-5p mimic ⑤ $\text{CAFs}^{\text{sh-hsa\_circ\_0056686}}$-CM+NC inhibitor

⑥ $\text{CAFs}^{\text{sh-hsa\_circ\_0056686-CM}}$+miR-515-5p inhibitor

Figure 7D

ATF6

① ② ③ ④ ⑤ ⑥

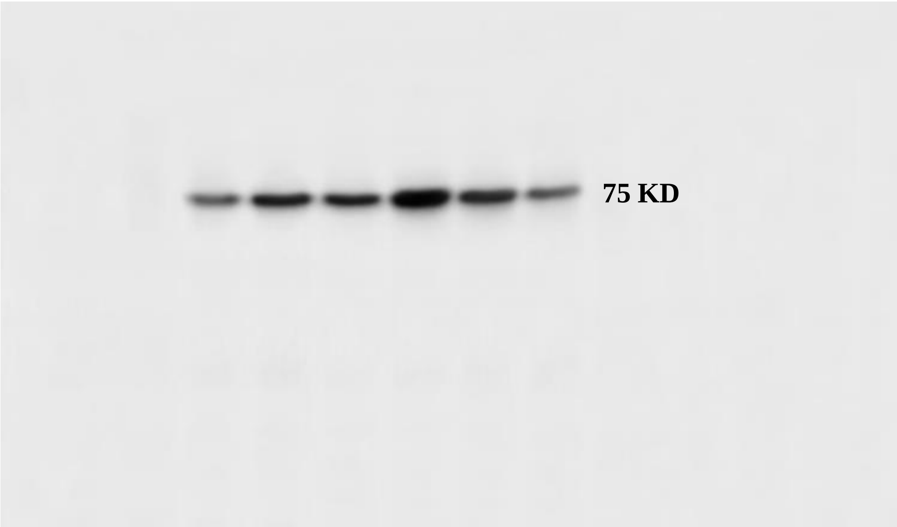

① $\text{CAFs}^{\text{sh-NC}}$-CM ② $\text{CAFs}^{\text{sh-hsa\_circ\_0056686}}$-CM ③ $\text{CAFs}^{\text{sh-hsa\_circ\_0056686}}$-CM+NC mimic

④ $\text{CAFs}^{\text{sh-hsa\_circ\_0056686-CM}}$+miR-515-5p mimic ⑤ $\text{CAFs}^{\text{sh-hsa\_circ\_0056686}}$-CM+NC inhibitor

⑥ $\text{CAFs}^{\text{sh-hsa\_circ\_0056686-CM}}$+miR-515-5p inhibitor

Figure 7D

GAPDH

① ② ③ ④ ⑤ ⑥

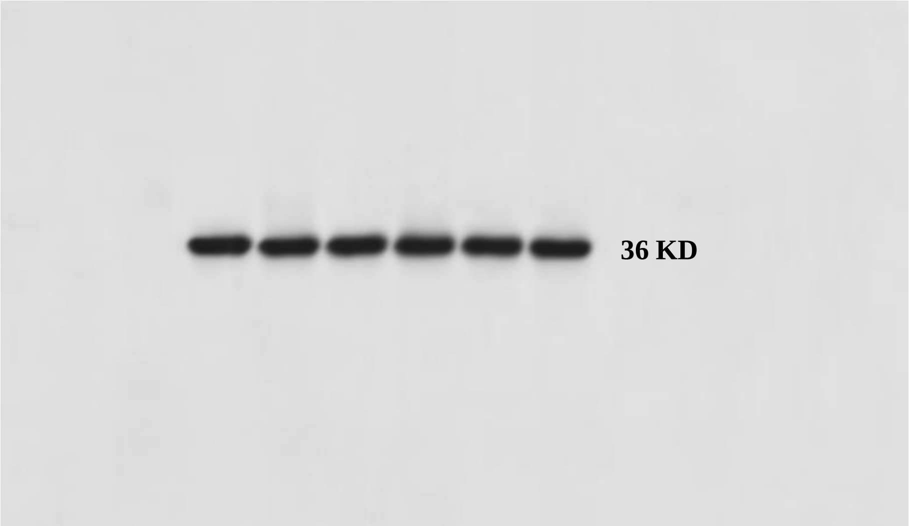

① CAFs$^{\text{sh-NC}}$-CM ② CAFs$^{\text{sh-hsa_circ_0056686}}$-CM ③ CAFs$^{\text{sh-hsa_circ_0056686}}$-CM+NC mimic

④ CAFs$^{\text{sh-hsa_circ_0056686-CM}}$+miR-515-5p mimic ⑤ CAFs$^{\text{sh-hsa_circ_0056686}}$-CM+NC inhibitor

⑥ CAFs$^{\text{sh-hsa_circ_0056686-CM}}$+miR-515-5p inhibitor

Figure 7E

COL1A1

① ② ③ ④ ⑤ ⑥

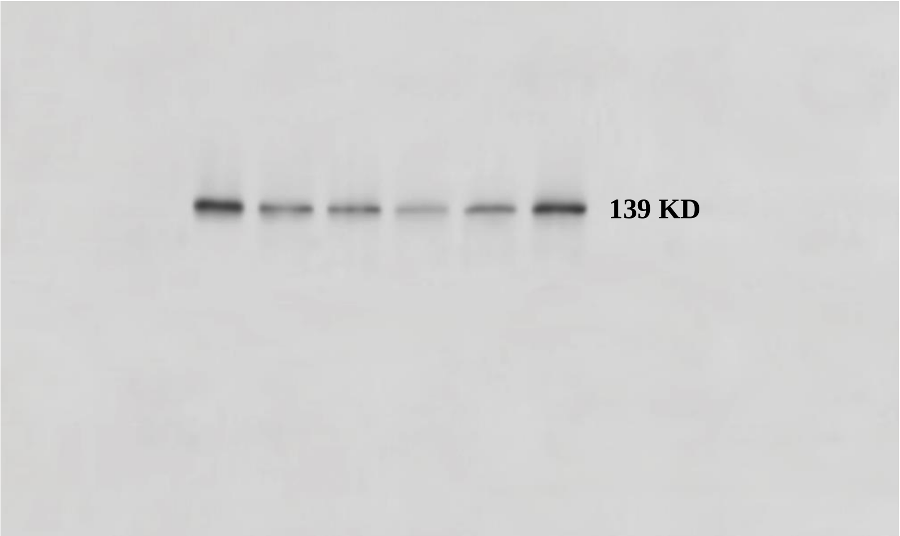

① CAFs$^{\text{sh-NC}}$-CM ② CAFs$^{\text{sh-hsa_circ_0056686}}$-CM ③ CAFs$^{\text{sh-hsa_circ_0056686}}$-CM+NC mimic

④ CAFs$^{\text{sh-hsa_circ_0056686-CM}}$+miR-515-5p mimic ⑤ CAFs$^{\text{sh-hsa_circ_0056686}}$-CM+NC inhibitor

⑥ CAFs$^{\text{sh-hsa_circ_0056686-CM}}$+miR-515-5p inhibitor

Figure 7E

COL1A2

① ② ③ ④ ⑤ ⑥

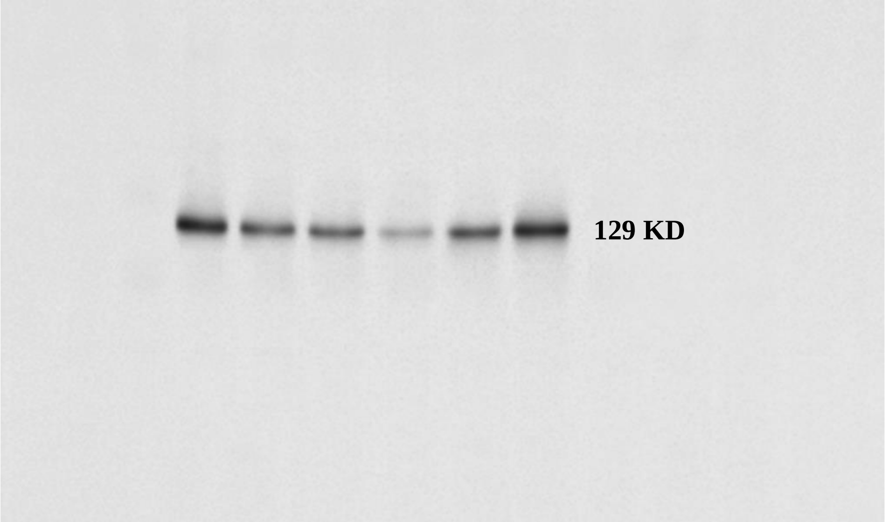

① CAFs$^{sh\text{-}NC}$-CM ② CAFs$^{sh\text{-}hsa_circ_0056686}$-CM ③ CAFs$^{sh\text{-}hsa_circ_0056686}$-CM+NC mimic

④ CAFs$^{sh\text{-}hsa_circ_0056686\text{-}CM}$+miR-515-5p mimic ⑤ CAFs$^{sh\text{-}hsa_circ_0056686}$-CM+NC inhibitor

⑥ CAFs$^{sh\text{-}hsa_circ_0056686\text{-}CM}$+miR-515-5p inhibitor

Figure 7E

COL3A1

① ② ③ ④ ⑤ ⑥

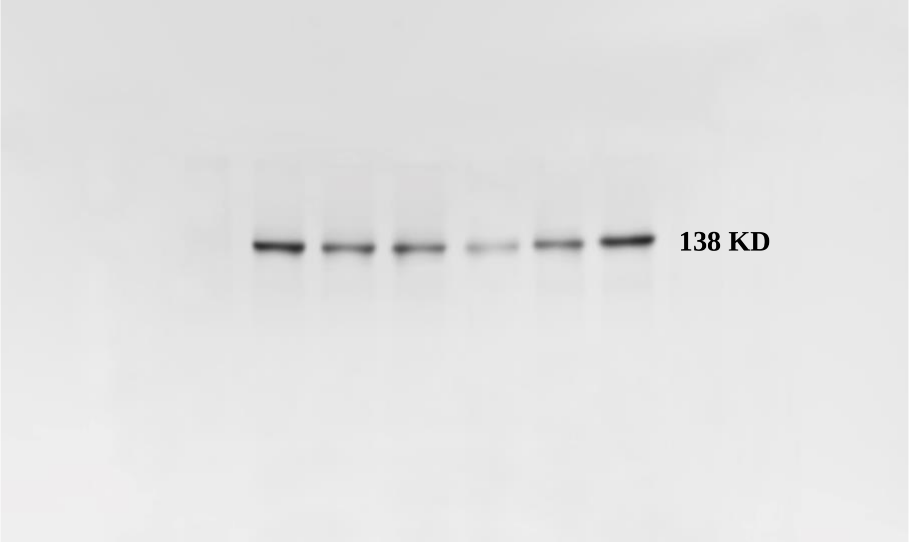

① CAFs$^{sh\text{-}NC}$-CM ② CAFs$^{sh\text{-}hsa_circ_0056686}$-CM ③ CAFs$^{sh\text{-}hsa_circ_0056686}$-CM+NC mimic

④ CAFs$^{sh\text{-}hsa_circ_0056686\text{-}CM}$+miR-515-5p mimic ⑤ CAFs$^{sh\text{-}hsa_circ_0056686}$-CM+NC inhibitor

⑥ **CAFs$^{\text{sh-hsa_circ_0056686-CM}}$+miR-515-5p inhibitor**

Figure 7E

GAPDH

① ② ③ ④ ⑤ ⑥

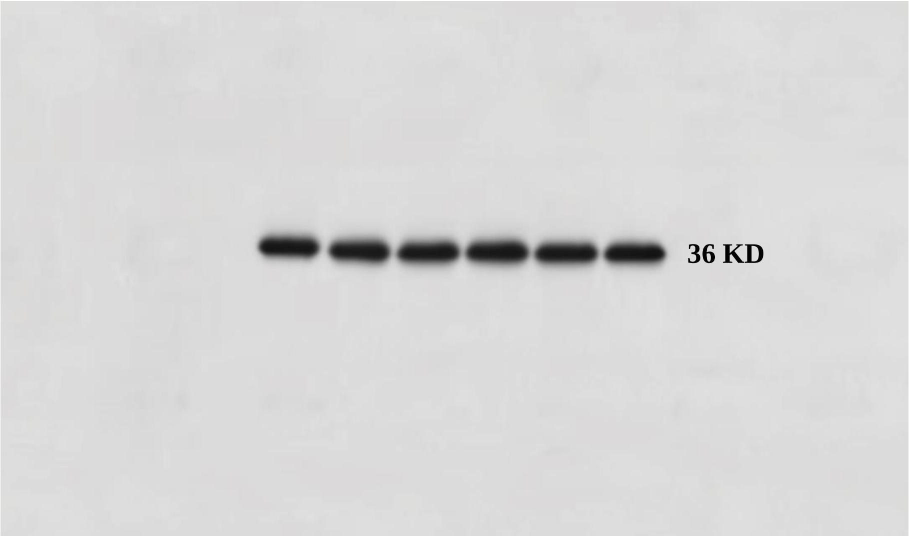

① **CAFs$^{\text{sh-NC}}$-CM** ② **CAFs$^{\text{sh-hsa_circ_0056686}}$-CM** ③ **CAFs$^{\text{sh-hsa_circ_0056686}}$-CM+NC mimic**

④ **CAFs$^{\text{sh-hsa_circ_0056686-CM}}$+miR-515-5p mimic** ⑤ **CAFs$^{\text{sh-hsa_circ_0056686}}$-CM+NC inhibitor**

⑥ **CAFs$^{\text{sh-hsa_circ_0056686-CM}}$+miR-515-5p inhibitor**
