# Supplementary material for: Hsa_circ_0056686, derived from cancer-associated fibroblasts, promotes cell proliferation and suppresses apoptosis in uterine leiomyoma through inhibiting endoplasmic reticulum stress
Source: PLoS One. 2022 Apr 7;17(4):e0266374. doi: 10.1371/journal.pone.0266374 (PMC8989227; doi:10.1371/journal.pone.0266374)
Supplement: Attachment — Submitted filename: Response to Reviewers.docx [file pone.0266374.s003.docx]

Dear Editor,

Thanks for your letter. We highly appreciate the valuable comments raised by the reviewers on our manuscript entitled " Hsa_circ_0056686, derived from cancer-associated fibroblasts, promotes cell proliferation and suppresses apoptosis in uterine leiomyoma through inhibiting endoplasmic reticulum stress " (ID: PONE-D-21-23535). We have improved our manuscript according to the comments, and the amendments are highlighted in red in the revised manuscript. Point to point responses are listed below. We would like to resubmit our manuscript for your kind consideration.

Thanks again.

Yours sincerely,

Airong Zhang

Department of Medical Laboratory, Zhumadian Central Hospital, Zhumadian, China.

Email: [airongz@21cn.com](mailto:airongz@21cn.com)

Although the reviewer feels that the study is potentially interesting, the reviewer requests the authors to address several concerns before publication. In addition, I also have several concerns. Specifically, the authors need to show the percentages of cells in each quadrant of the flow cytometry in Fig. 1d, 3d, 5b, 7a. Ideally, the authors should show the mean +/- SD of triplicate samples and determine the statistical significance of percentages of apoptotic cells in each condition. It is unclear why LSMCs spontaneously develop ER stress and undergo apoptosis. Moreover, it is also unclear why the conditioned medium of CAFs attenuates the expression of GPR78, CHOP, and ATF6 in LSMCs. To investigate the mechanisms underlying these issues may beyond the scope of the present study, however, the authors should discuss these points in more detail.

**Response:** Good comments. We added the percentage of cells in each quadrant of flow cytometry in figs 1D, 3D, 5B, 7A.

In this study，the data from at least three replicate experiments were presented as mean ± SD. We modified the poorly described sentences and added more details of statistical analysis in the materials and methods section (Statistical Analysis).

As we know, ER stress signaling in the tumor microenvironment and cancer associated fibroblasts (CAFs) play important roles in tumor progression, and common drivers of ER stress are often low oxygen, nutrients, low pH, and reactive oxygen species accumulation. We fully understand the reviewer′s concerns. In fact, there is some degree of ER stress and apoptosis in cultured cells without any stress (maybe culture in vitro itself is a “stress factor”), and similar results have been reported in previous studies **(****including but not just Ref.1 &** **Ref.2)**.

**Ref.1:** Tumour suppressor candidate 3 inhibits biological function and increases endoplasmic reticulum stress of melanoma cells WM451 by regulating AKT/GSK3-β/β-catenin pathway. doi: 10.1002/cbf.3515.

**Ref.2:** MSRB3 promotes the progression of clear cell renal cell carcinoma via regulating endoplasmic reticulum stress. doi: 10.1016/j.prp.2019.152780.

In recent years, several studies have found that CAFs are tightly linked to ER stress in tumor progression. Zeng et al. found that curcumin induced apoptosis and cell cycle arrest of CAFs, which were mainly caused by ROS mediated ER stress pathway, and mechanistically, curcumin induced upregulation of ROS via PERK-eIF2α-ATF4 axis triggers ER stress in CAFs **(****Ref.3)**. We fully consider the reviewers' concerns. Indeed, in a variety of tumor progressions, CAFs function as tumor promoting cellular activities, and inhibition of the ER stress pathway is an important mechanism of immune evasion in cancer cells. Therefore, we believe that CAFs could inhibit ER stress in tumor cells to some extent, and the two synergistically promote tumor progression. However, the specific regulatory mechanisms between tumor cells, CAFs and ER stress exceed the scope of this study, and still need further in-depth investigation. We added new literature in the discussion section and discussed the limitations of this study.

**Ref.3:** Curcumin promotes cancer-associated fibroblasts apoptosis via ROS-mediated endoplasmic reticulum stress. doi: 10.1016/j.abb.2020.108613.

2. We note that the grant information you provided in the ‘Funding Information’ and ‘Financial Disclosure’ sections do not match.

When you resubmit, please ensure that you provide the correct grant numbers for the awards you received for your study in the ‘Funding Information’ section.

**Response:** Thanks. We collated and revised funding information for this study.

Reviewers' comments:

Reviewer's Responses to Questions

Comments to the Author

1. Is the manuscript technically sound, and do the data support the conclusions?

The manuscript must describe a technically sound piece of scientific research with data that supports the conclusions. Experiments must have been conducted rigorously, with appropriate controls, replication, and sample sizes. The conclusions must be drawn appropriately based on the data presented.

Reviewer #1: Yes

2. Has the statistical analysis been performed appropriately and rigorously?

Reviewer #1: Yes

3. Have the authors made all data underlying the findings in their manuscript fully available?

The PLOS Data policy requires authors to make all data underlying the findings described in their manuscript fully available without restriction, with rare exception (please refer to the Data Availability Statement in the manuscript PDF file). The data should be provided as part of the manuscript or its supporting information, or deposited to a public repository. For example, in addition to summary statistics, the data points behind means, medians and variance measures should be available. If there are restrictions on publicly sharing data-e.g., participant privacy or use of data from a third party—those must be specified.

Reviewer #1: Yes

4. Is the manuscript presented in an intelligible fashion and written in standard English?

PLOS ONE does not copyedit accepted manuscripts, so the language in submitted articles must be clear, correct, and unambiguous. Any typographical or grammatical errors should be corrected at revision, so please note any specific errors here.

Reviewer #1: Yes

5. Review Comments to the Author

Please use the space provided to explain your answers to the questions above. You may also include additional comments for the author, including concerns about dual publication, research ethics, or publication ethics. (Please upload your review as an attachment if it exceeds 20,000 characters)

Reviewer #1: The authors demonstrated that hsa_circ_0056686 was up-regulated in CAFs around uterine leiomyoma, and influenced the CAFs-mediated proliferation, migration and apoptosis of ULM cells. They also showed that hsa_circ_0056686 directly bound with miR-515-5p and this bounding contributed to the influence on the CAFs-mediated proliferation, migration, and apoptosis.

This study is potentially interesting; however, some issues need to be addressed.

Listed below are my specific concerns and suggestions:

1. The effect of hsa_circ_0056686 on the CAFs-mediated proliferation, migration and apoptosis of ULM cells seems to be totally dependent on miR-515-5p. The data examining effects of the miR-515-5p antagomir on the CAFs-mediated proliferation, migration and apoptosis would strengthen the author’s claim.

**Response:** Good comments. The miR-515-5p mimic and miR-515-5p inhibitor were transfected into ULM cells mediated by CAFs, respectively. The results showed that overexpression of miR-515-5p inhibited cell migration and promoted cell apoptosis compared with CAFs cells transfected with sh-hsa_circ_0056686. In addition, overexpression of miR-515-5p promoted cleaved caspase3, GRP78, CHOP and ATF6 protein expression, inhibited Bcl-2, COL1A1, COL1A2 and COL1A3 protein expression, and transfection of miR-515-5p inhibitor to cells had the opposite effect as transfection of miR-515-5p mimic. The specific results were presented in Fig. 7 and results 7.

2. The authors proposed AKAP13 as a possible target of miR-515-5p. Are there any previous reports or data that demonstrated the contribution of AKAP13 to proliferation, migration and apoptosis?

**Response:** Good comments. In this study, we predicted that AKAP13 was a potential target of miR-515-5p through online bioinformatics databases, and although it has not been clarified in this study whether miR-515-5p plays a regulatory role on uterine fibroid progression via AKAP13, it will be an important target for our subsequent study. In a previous study, Ng et al. found that AKAP13 was overexpressed in uterine fibroid tissue **(****Ref 1)**, which set the stage for our subsequent studies. In addition, another study claimed that AKAP13 expression knockdown suppressed the proliferation and invasion of acute myeloid leukaemia cells **(****Ref 2)**. We added the corresponding literature in the discussion section.

**Ref 1:** A-Kinase Anchoring Protein 13 (AKAP13) Augments Progesterone Signaling in Uterine Fibroid Cells. J Clin Endocrinol Metab. doi: 10.1210/jc.2018-01216.

**Ref 2:** Long non-coding RNA ZNF667-AS1 knockdown curbs liver metastasis in acute myeloid leukemia by regulating the microRNA-206/AKAP13 Axis. doi: 10.2147/CMAR.S269258.

3. In figure 4B, the authors should demonstrate the western blotting data using anti-‘cleaved’ caspase 3 antibody.

**Response:** Thanks. In fact, the antibody used in the experiments was a cleaved caspase 3 antibody (in China, we verbally called it caspase 3 as brief), and we corrected its name in Figure 4B.

4. Add a molecular weight marker to all western blot images in this manuscript.

**Response:** Thanks. We labeled the molecular weights of the proteins in all Western blot images.

5. More thorough explanations and important details on the experimental procedures is missing throughout the paper. In particular, effort should be performed for clarifying method to detect the protein amounts of collagens and the expression level of hsa_circ_0056686.

**Response:** Thanks. We carefully checked and revised the manuscript and added more details of experimental procedures in the materials and methods section.

6. PLOS authors have the option to publish the peer review history of their article (what does this mean?). If published, this will include your full peer review and any attached files.

If you choose “no”, your identity will remain anonymous but your review may still be made public.

Do you want your identity to be public for this peer review? For information about this choice, including consent withdrawal, please see our Privacy Policy.

Reviewer #1: No

We appreciate for Editors/Reviewers’ warm work earnestly, and hope that the correction will meet with approval.

Once again, thank you very much for your comments and suggestions.
