# Supplementary material for: Hsa_circ_0056686, derived from cancer-associated fibroblasts, promotes cell proliferation and suppresses apoptosis in uterine leiomyoma through inhibiting endoplasmic reticulum stress
Source: PLoS One. 2022 Apr 7;17(4):e0266374. doi: 10.1371/journal.pone.0266374 (PMC8989227; doi:10.1371/journal.pone.0266374)
Supplement: Attachment — Submitted filename: Response to Reviewers.docx [file pone.0266374.s004.docx]

Dear Editor,

Thanks for your letter. We highly appreciate the valuable comments raised by the reviewers on our manuscript entitled " Hsa_circ_0056686, derived from cancer-associated fibroblasts, promotes cell proliferation and suppresses apoptosis in uterine leiomyoma through inhibiting endoplasmic reticulum stress " (ID: PONE-D-21-23535R1). We have improved our manuscript according to the comments, and the amendments are highlighted in red in the revised manuscript. Point to point responses are listed below. We would like to resubmit our manuscript for your kind consideration.

Thanks again.

Yours sincerely,

Airong Zhang

Department of Medical Laboratory, Zhumadian Central Hospital, Zhumadian, China.

Email: [airongz@21cn.com](mailto:airongz@21cn.com)

The reviewer has been satisfied with your revision, however, the reviewer still has minor concerns. Specifically, although the authors added the molecular weight markers in Figures, there is no molecular weight markers in Supplementary Figures. Moreover, the order of Figures are not correct; Figure 1 appears to be placed after Figure 7. Thus, I would like to recommend that the authors respond to these points raised by the reviewers.

**Response:** Sorry for our carelessness. Fist, we added the molecular weight markers in Supplementary Figures. In addition, we have adapted the order of figures 1 and 7 when uploading our latest revised manuscript.

2. Thank you for stating the following financial disclosure:

"No

Conceptualization: Meifang, Airong Zhang.

Data curation: Meifang, Zhichen Lin, Dongfang Guo, Airong Zhang.

Formal analysis: Meifang, Airong Zhang.

Funding acquisition: Meifang, Airong Zhang.

Methodology: Meifang, Airong Zhang.

Investigation: Zhichen Lin, Dongfang Guo, Airong Zhang.

Project administration: Meifang, Airong Zhang.

Resources: Meifang, Zhichen Lin, Dongfang Guo, Airong Zhang.

Supervision: Airong Zhang.

Writing - original draft: Meifang, Zhichen Lin, Dongfang Guo, Airong Zhang.

Writing - original draft: Meifang, Zhichen Lin, Dongfang Guo, Airong Zhang."

At this time, please address the following queries:

a) Please clarify the sources of funding (financial or material support) for your study. List the grants or organizations that supported your study, including funding received from your institution.

b) State what role the funders took in the study. If the funders had no role in your study, please state: “The funders had no role in study design, data collection and analysis, decision to publish, or preparation of the manuscript.”

c) If any authors received a salary from any of your funders, please state which authors and which funders.

d) If you did not receive any funding for this study, please state: “The authors received no specific funding for this work.”

**Response:** Thanks. We collated and revised financial disclosures for this study. Details are as follows,

“**Authors' contributions**

**Conceptualization:** Meifang, Airong Zhang.

**Data curation:** Meifang, Zhichen Lin, Dongfang Guo, Airong Zhang.

**Formal analysis:** Meifang, Airong Zhang.

**Methodology:** Meifang, Airong Zhang.

**Investigation:** Zhichen Lin, Dongfang Guo, Airong Zhang.

**Project administration:** Meifang, Airong Zhang.

**Resources:** Meifang, Zhichen Lin, Dongfang Guo, Airong Zhang.

**Supervision:** Airong Zhang.

**Writing - original draft:** Meifang, Zhichen Lin, Dongfang Guo, Airong Zhang.

**Writing - original review & editing:** Meifang, Zhichen Lin, Dongfang Guo, Airong Zhang.

**Funding**

The author(s) received no specific funding for this work.”

Reviewers' comments:

Reviewer's Responses to Questions

Comments to the Author

1. If the authors have adequately addressed your comments raised in a previous round of review and you feel that this manuscript is now acceptable for publication, you may indicate that here to bypass the “Comments to the Author” section, enter your conflict of interest statement in the “Confidential to Editor” section, and submit your "Accept" recommendation.

Reviewer #1: (No Response)

2. Is the manuscript technically sound, and do the data support the conclusions?

The manuscript must describe a technically sound piece of scientific research with data that supports the conclusions. Experiments must have been conducted rigorously, with appropriate controls, replication, and sample sizes. The conclusions must be drawn appropriately based on the data presented.

Reviewer #1: Yes

3. Has the statistical analysis been performed appropriately and rigorously?

Reviewer #1: Yes

4. Have the authors made all data underlying the findings in their manuscript fully available?

The PLOS Data policy requires authors to make all data underlying the findings described in their manuscript fully available without restriction, with rare exception (please refer to the Data Availability Statement in the manuscript PDF file). The data should be provided as part of the manuscript or its supporting information, or deposited to a public repository. For example, in addition to summary statistics, the data points behind means, medians and variance measures should be available. If there are restrictions on publicly sharing data—e.g. participant privacy or use of data from a third party—those must be specified.

Reviewer #1: Yes

5. Is the manuscript presented in an intelligible fashion and written in standard English?

PLOS ONE does not copyedit accepted manuscripts, so the language in submitted articles must be clear, correct, and unambiguous. Any typographical or grammatical errors should be corrected at revision, so please note any specific errors here.

Reviewer #1: Yes

6. Review Comments to the Author

Please use the space provided to explain your answers to the questions above. You may also include additional comments for the author, including concerns about dual publication, research ethics, or publication ethics. (Please upload your review as an attachment if it exceeds 20,000 characters)

Reviewer #1: The authors did not answer my comment #4 'Add a molecular weight marker to all western blot images.'

**Response:** Sorry for our carelessness. We added the molecular weight markers in Supplementary Figures of latest revised manuscript.

7. PLOS authors have the option to publish the peer review history of their article (what does this mean?). If published, this will include your full peer review and any attached files.

If you choose “no”, your identity will remain anonymous but your review may still be made public.

Do you want your identity to be public for this peer review? For information about this choice, including consent withdrawal, please see our Privacy Policy.

Reviewer #1: No

We appreciate for Editors/Reviewers’ warm work earnestly, and hope that the correction will meet with approval.

Once again, thank you very much for your comments and suggestions.
